# Biobank@VITO: Biobanking the General Population in Flanders

**DOI:** 10.3389/fmed.2020.00037

**Published:** 2020-02-14

**Authors:** Rosette Van Den Heuvel, Elly Den Hond, Ann Colles, Vera Nelen, Karen Van Campenhout, Greet Schoeters

**Affiliations:** ^1^Unit Health, Flanders' Research and Technology Organisation on Cleantech and Sustainable Development (VITO), Mol, Belgium; ^2^Department of Environment, Provincial Institute for Hygiene (PIH), Antwerp, Belgium; ^3^Vlaams Planbureau voor Omgeving (VPO), Flemish Government, Brussels, Belgium

**Keywords:** population biobank, human biomonitoring, biobank, FLEHS, 3XG

## Abstract

During the last 15 years, VITO has established an infrastructure for biobanking a collection of biological samples from the general population in Flanders (Belgium). This biobank was set up to contribute to future, yet unspecified, research questions in the field of environment and health. Biobank@VITO is a population biobank in which bio-specimen including human peripheral blood, cord blood, and blood derivatives (e.g., serum, plasma, cells, RNA, DNA), urine, hair, nails, exhaled breath condensate, saliva DNA, and human breast milk collected from non-diseased populations are preserved. Currently, the biobank stores about 70,000 samples from 7,700 individuals. These biospecimen were collected since 2002 in different human biomonitoring studies comprising European (e.g., DEMOCOPHES, HBM4EU), national (e.g., WHO human breastmilk studies), Flemish (Flemish Environment and Health Study (FLEHS) campaigns), and local (e.g., hotspots, 3xG project) well-defined and ethically approved research projects. Participants to the surveys included different age groups (newborns, children, adolescents, and adults) and were representatively selected with regard to gender, age class, residence, and/or socioeconomic status (SES). In each campaign, samples were stored in the Biobank@VITO. The registration, preservation, and management of the samples in the biobank were done in a qualitative and uniform manner which guarantees the traceability of all samples. The samples in the biobank have an extended information backbone on the lifestyle, environment, and health status of the donor. The biological samples in the biobank are an invaluable archive that can be used to address specific policy and research questions in the future, to test old samples with new technology and according to the latest methods and insights or to measure newly identified pollutants in old samples looking for long-term trends.

## Introduction

Flanders is generally considered to be one of Europe's economic top regions with an extensive transportation network and intensive industrial activity. At the same time, Flanders is one of the most densely populated areas of Europe. Exposure to traffic or industrial emissions remain an important factor for adverse human health effects. Also lifestyle, diet, socio-economic, or physical exercise have been shown to have an impact on the health of the population. All these issues clearly illustrate that the relationship between environmental quality, socio-economic living conditions, individual behavior, and public health is a very complex one. As a response to this and to the societal challenges, human biomonitoring data and auxiliary personal information are collected and used as science based evidence to underpin measures for safeguarding environmental quality and to minimize the adverse effects of environmental stressors. In 2003, the Flemish government voted the Decree on Preventive Health Care as a legal recognition of environmental health in which the Flemish government imposes itself to perform human biomonitoring (HBM), i.e., the measurement of potentially adverse chemicals in human matrices such as urine or blood. In order to achieve a structured and coordinated approach for human biomonitoring in Flanders, the Flemish Centre of Expertise on Environment and Health was founded by the Flemish government (Department of Economics, Science and Innovation; Flemish Agency for Care and Health; and Department of Environment, Nature and Energy). The Center is the main driving force behind the Flemish Environment and Health Studies, FLEHS. Human biomonitoring is the core of the programme on which several research projects are engrafted. Internal concentrations of a broad range of environmental chemicals and/or associated health effects are monitored. Exposure biomarkers provide information on the internal exposure to chemical substances and effect biomarkers provide information on the biological consequences of the presence of these chemical substances in the human body.

For over 15 years, a large number of human samples (urine, blood, plasma, hair,…) have been collected in the Flemish general population and have been analyzed for the presence of environmental chemicals or their metabolites. This allowed to estimate population reference values (statistically derived numbers that indicate the upper margin of background exposure to a given substance in a defined population at a given time) of exposure to both well-established and new or emerging chemicals (1). Also other projects, typically addressing specific human health issues around areas with specific environmental pressure (hot-spots), or topics of societal concern, use HBM for mapping and benchmarking the levels of potentially adverse chemicals in the otherwise healthy general population (2). In each campaign, additionally to biomarker analyses which were planned to answer the research questions, extra samples were taken and stored in the biobank. These samples may be used in the future for both prospective and retrospective research. The concept of the biobank may be considered as a biological archive of internal human exposure levels in Flanders, and as such allows analysis of the past with future technologies.

This paper describes the mission, the objectives, the management, and the sample collections of the population biobank hosted at VITO.

## Mission

Human biomonitoring in the general population is an intrinsic part of the Flemish approach to science based policy-making in the field of environment and health. The monitoring programs of the Flemish Centre of Expertise for Environment and Health comprise now already a large number of chemicals. However, due to the rapid progress of technology and predicted increases in production volume of chemicals over the next 30 years, we expect increased exposure through environment and use of consumption products and people will come into contact with ever more and new foreign substances. As a result, additional relevant research or policy questions may arise in the future. Therefore, as part of the FLEHS programme and on request of the Flemish authorities, it was decided to build a human biobank in which samples are stored for potential use in the future.

Since 2002, biobank samples were stored in a biorepository. The human samples originate from members of different age groups from the Flemish population that were invited to participate in the studies according to a randomized two stage recruitment strategy and that gave permission to take and store their samples in the biobank. A large number of human biological samples including blood and blood derivatives, urine, hair, nails, exhaled breath condensate, saliva DNA, and human breast milk samples have been collected over the years, resulting from the activities of the Flemish Centre of Expertise for Environment and Health and a number of other biomonitoring activities. The samples are well-documented in terms of individual exposure to environmental chemicals and related health status. Additionally, extensive information on lifestyle, personal characteristics, food consumption, individual risk perception, etc., is available from self-assessed questionnaires at the time of sampling. In this way, the samples are invaluable for new knowledge acquisition in the field of environment and health. In order to preserve this potential optimally for the future and to be able to serve for further research, a well-developed biobank is necessary.

Biobank@VITO aims at setting up a professional, state-of-the-art biobanking structure in which the focus is put on collecting samples of the general population. The goal is 2-fold: (1) provide a facility where the collection of human samples, gathered over the last two decades, are stored under state-of-the-art circumstances; (2) provide a professional storage facility for future (prospective) biomonitoring initiatives in Flanders and elsewhere. Both objectives are implemented with attention to respect the privacy of the participants [GDPR (General Data Protection Regulation) compliant] and ethical aspects.

## Objectives

### Additional Measurements to Extend Studies

The biobank allows additional biomarker measurements to expand past and ongoing studies. Supplementary biomarker analyses (both biomarkers of exposure and biomarkers of effect) can be carried out at a later time in an existing cohort of which much information is already available. This saves a lot of costs and work as no new recruitment and sampling should be organized, and maximum use can be made of the questionnaire data and measurements that are already available in the database.

With appropriate storage of samples, retrospective analyses of samples from years or even decades ago can be performed using state-of-the-art analytical technology to assess internal exposure and associated biological effects.

### Follow Trends in Time

Following time trends requires different campaigns that are spread over time. The biobank allows screening of new and emerging chemicals in various matrices, once appropriate biomarkers are developed. For these new biomarkers, samples from the past can then be analyzed to check the level of these substances in the body x years ago and to assess how levels evolve over time. These analyzes can be performed on pooled samples as well as on individual samples. Such time trends can best be followed up in reference populations.

By repeating biomonitoring campaigns at regular intervals, human exposure to pollutants over time can be monitored and policy measures can be evaluated (3).

### Prospective Studies

Increasingly, results of prospective cohort studies are used in environmental health research. Repetitive sampling in the same individuals is extremely powerful to unravel the often very subtle and complex relationships between environmental (chemical or lifestyle related) stressors and potentially adverse health effects. Follow-up studies allow people to be monitored during successive cycles with a specific pre-defined goal in order to study health effects in the longer term (years). Therefore, questionnaires and additional examinations (e.g., including new blood or urine collections) can be scheduled at regular intervals as well as requesting personal health data from registers. By submitting samples to the biobank at each of the successive cycles, new research hypotheses can be tested afterwards.

Often, these prospective studies are birth cohorts, in which the child is followed from the moment of birth (or already *in utero*) for several years or even decades. Prospective cohorts like the 3xG study, or the various newborn cohorts of the FLEHS cycles (4) are examples of ongoing longitudinal birth cohorts studies in Flanders.

### Retrospective Studies

Samples that are stored in the biobank, may be used for retrospective assessment of both exposure and biological effects as new and improved technical innovations become available or to give answer to new research questions. Samples from follow-up studies that were stored under the appropriate conditions could be used for (1) the detection of novel effect biomarkers (e.g., omics) to monitor the early onset of diseases or (2) the identification of historical exposure that is involved in the onset of diseases later in life The availability of biobank samples in combination with extensive information on the participants allows to design nested case-control studies, and hence offers reductions in costs and efforts of data collection and works more efficiently in case of rare outcomes, expensive measurements, or missing covariates.

## Privacy and Ethical Aspects

Since human biomonitoring campaigns involve processing of personal data, the studies were registered at the Belgian Privacy Commission (CBPL = Commissie voor de bescherming van de persoonlijke levenssfeer) until the new GDPR law came into force on 25 May 2018 [(EU) 2016/679]. Following this, the new guidelines on the protection of personal data were applied.

In addition, attention is paid to compliance with the ethical code for dealing with biological material (5). All samples present in the Biobank@VITO have been collected in the context of a predefined research project. Each human biomonitoring study was submitted for approval to the Ethics Committee of the university of Antwerp for all studies, and additionally to the Ethics Committee of local hospitals in some specific cases (e.g., newborn studies). The initial principle of the biobank, being the storage of samples in the long term, was explained to the participant at the start of the study in the information brochure and in the consent form. The study participant gave written and signed permission for the storage of biological samples in the biorepository. In addition, contact details of the principal investigator (PI) and responsible doctor were given on the information letter and consent form (template forms in Supplementary Material). Participants gave donor consent which means that the rationale for the use of donors' samples and data is explained in great detail in the consent form. In mother-birth cohorts, the participating mothers filled out an informed consent. In case minor children were involved, parents signed the informed consent on behalf of the children. In the adolescent population (14–16 years), both the minor participant and one of the parents gave written informed consent to participate in the study. In the case children reach the age of majority, they will be asked to re-consent for the storage and use of their samples collected at the minor age.

No names or addresses of participants are registered in the Biobank@VITO. All participants, their samples and additional information are pseudonymized by the use of a unique identification code. The key to this code is only known to the field work team that works under the supervision of the responsible medical doctor. All communication with the participants takes place through the latter. The participant can request at any time information about the state of the research and the samples in the biobank, either through the PI or the medical doctor, but only the responsible study doctor can report back to the participant. Each individual can terminate further participation in the study and/or request at each moment to destroy his/her remaining personal samples. The results that are already in the database remain available for the researchers unless the participant explicitly asks to remove all his/her data. The data manager of the field work team contacts the Biobank manager if the consent status of the participant changes. The Biobank manager takes the necessary actions based on the unique identification code. For specific subgroups, permission from the participant was obtained to request personal health data from registers, e.g., data from child care services in the newborn group, data from school health investigations in the adolescent group, etc. This information is requested by the medical doctor, and can be coupled to the central study database on the basis of the unique identification code.

The participants are aware of the fact that the analyses that are performed on biobank samples are not communicated on an individual level, since this is explicitly mentioned in the informed consent. However, summary reports of the studies that are performed on biobank samples are communicated via the study websites. Based on these reports, participant can follow the new analyses that are performed. At any moment, a participant has the right to request his/her personal results via the PI or the medical doctor, and this information will then by shared by the medical doctor, either via letter or via telephone, to be able to provide the necessary background information. Also in case of an alarming result for a health parameter that is clearly interpretable, the medical doctor could communicate this result directly to the participant, after discussion and consent by the management board.

## Methods

### Sample Management

The human samples are collected, registered, stored, and managed in a qualitative and uniform manner. Procedures for sample collection, sample pre-treatment, aliquoting, primary and secondary sample tubes, temporary storage, transport conditions, and storage method are documented in a detailed manual. Biobank@VITO uses an unambiguous donor identification system which guarantees the traceability of all samples at any time. All samples are uniformly provided with a unique label number. The sample number is linked to a minimal dataset such as the identification code of the participant, collection date, sample type, sample volume, tube code, and sample location. All this information is stored in a database. Since 2015, a computer-based inventory LIMS (Laboratory Information Management System) system is in use for sample registration and management (Labvantage). The inventory enables to identify the location of any sample at all times.

Sample types include whole (cord)blood, (cord)blood derivatives such plasma, serum, red blood cells, white blood cells, RNA and DNA, urine, hair, nails, saliva DNA, exhaled breath condensate, and breastmilk. Samples are stored under optimal conditions in storage facilities at various storage temperatures including −80°C, −20°C and room temperature.

**Figure 1:**
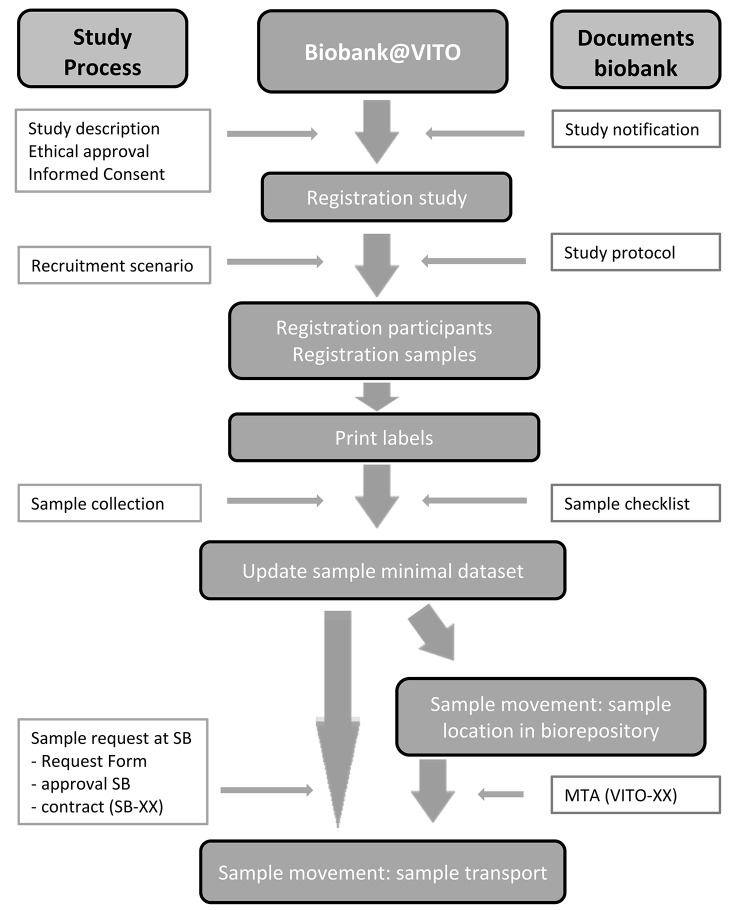
Management structure Biobank@VITO and sample flow chart. (SB, Supervisory Board of the Centre of Expertise on Environment and Health; MTA, Material Transfer Agreement).

Continuously efforts are made to improve the quality management system of the biobank in order to be compliant with OECD and ISO guidelines for biobanks (6, 7) and the Belgian law on biobanking (8). Biobank@VITO received ethical approval and was notified to FAMHP (Federal Agency for Medicines and Health Products) (notification number BB190064). The organizational structure of Biobank@VITO and the sample flow in the biobank are shown in Figure 1. The workflow starts with the notification of a new study at the biobank manager. Proof of ethical review, study design and minimal dataset are needed to proceed the registration of samples in the sample management system (LIMS, Labvantage). Sample release can only take place if the necessary formalities are met, in particular signed MTA's (material transfer agreement).

### Quality Assurance Measures

The pre-analytic phase includes the collection, transport and registration of the samples. The different steps are described in detail in a scenario or in a specific procedure (SOPs). The management of material and equipment is described in a SOP and registrations are kept in a database.

Samples are stored under the most appropriate condition depending on the sample type and the biomarker to measure. The quality and the stability of the samples is checked on a regular basis and is dependent on the type of sample and the planned analyses e.g., DNA/RNA integrity check, biomarker stability using control samples kept under the same conditions as the actual samples. Samples are stored in small aliquots and freeze-thaw cycles are kept to a minimum. The storage time can be dependent on the quality of samples, on the number of freeze-thaw cycles, on the participants consent or can be a fixed period set by the PI.

Freezers and cooling systems are equipped with a computer-based temperature monitoring system for temperature control and an automatic alarm system in case of repository failure. Freezers are centralized in separate rooms that are not shared with other activities.

### Sample Access Policy

As participants gave donor consent and no broad consent, samples from collections are available for further research within the context of the well-defined research topic that researchers have had the sample donor's consent for. Therefore, novel usage of the samples considering any new research, further uses or new studies, is not possible without first obtaining a new consent of the donor. Supervision on the correct compliance with these terms is the responsibility of the Biobank manager.

In addition, Biobank@VITO hosts different samples collections and access to samples is dependent on the reuse policy of the collection's PI. Sample requests addressed directly to the Biobank@VITO will be referred to the relevant PI. As such, new research projects aiming to use samples from the Flemish Environment and Health Study (FLEHS) collections in the biobank have to apply a request to the Supervisory Board of the Centre of Expertise on Environment and Health. Data/sample transfer can only be carried out after approval by the Supervisory Board. Terms and conditions for data/sample request are defined by the Supervisory Committee. For every new analysis on biobank samples, the approval of an ethics committee is required.

## Collections

A large amount of samples have been collected from the general population of Belgium (e.g., DEMOCOPHES), Flanders (e.g., FLEHS, HBM4EU) or specific regions (e.g., hotspot studies, 3xG). These human biomonitoring studies have different scopes: they often combine specific research questions and policy-based goals, and are financed by different funders, such as the European Commission, the Flemish government, national, or regional organizations. An overview of the type and amount of samples in the biorepository which were collected in different studies is given in Table 1. The main studies are described in detail below.

**Table 1:** Collections of human biological material in Biobank@VITO.

**Study**	**Collection period**	**Population**	**Number participants**	**Gender (%) (male/female)**	**Sample types**	**Biobank samples *N***	**Storage**
FLEHS I	2002–2004	Newborns	1,196	52/48	Cord blood Cord blood plasma Cord bloodDNA	1,799 1,974 117	−20°C −20°C −80°C
	2003–2004	Adolescents (14–15 y)	1,679	53.1/46.9	Peripheral blood Serum Urine	1,636 1,445 1,688	−20°C −20°C −20°C
	2004–2005	Adults (50–65 y)	1,583	49/51	Peripheral blood Serum Urine	1,526 1,489 1,581	−20°C −20°C −20°C
FLEHS I birth cohort follow-up	2013–2014	Children (10 y)	133	33.1/66.9	Blood white blood cells Red blood cells Plasma Blood DNA Blood RNA Saliva DNA	99 300 86 134 100 132	−80°C −80°C −80°C −80°C −20°C −80°C
FLEHS II	2008–2009	Newborns	255	52/48	Cord blood plasma Cord blood cells Cord blood DNA	219 254 726	−80°C −80°C −80°C
	2008–2009	Adolescentsss (14–15 y)	210	57.6/42.4	Peripheral blood Serum Urine	196 64 1,828	−80°C −80°C −80°C/−20°C
	2008–2009	Adults (20–40 y)	204	47.1/52.9	Peripheral blood Serum Urine	406 622 2,033	−80°C −80°C −80°C/−20°C
FLEHS III	2014	Newborns	281	53.1/48.8	Cord blood Cord blood plasma Cord blood cells Hair mother Nails mother	1,710 122 279 196 159	−80°C/−20°C −80°C/−20°C −80°C RT RT
	2012–2013	Adolescentss (14–15 y)	208	45.7/54.3	Peripheral blood Peripheral blood cells Blood RNA Serum Plasma Urine EBC^*^	618 276 92 181 202 496 41	−80°C −80°C −20°C −80°C −80°C −80°C −80°C
	2014	Adults (50–65 y)	209	46/54	Peripheral blood Blood DNA Serum Urine	616 204 333 657	−80°C −20°C −80°C −80°C/−20°C
FLEHS IV + FLEHS I birth cohort (14–15 year)	2017–2018	Adolescents (14–15 y)	611	47.2/52.8	Peripheral blood Peripheral blood cells Blood RNA Serum Urine Hair	1,306 609 608 1,879 3,009 609	−80°C −80°C −20°C −80°C −80°C/−20°C RT
3XG	2011–2015	Mother–newborn cohort	301	50.8/19.2	Cord blood Cord blood cells Cord blood plasma Peripheral blood Serum Urine Breastmilk	1,039 1,110 930 1,211 721 5,575 196	−80°C/−20°C −80°C −80°C/−20°C −80°C −80°C/−20°C −80°C/−20°C −80°C/−20°C
DEMOCOPHES	2011–2012	Mother (≤45 y)—child cohort (6–11 y)	129	51.2/48.8	Urine Hair	873 263	−80°C RT
Hotspot study: Genk	2010	Adolescents (14–15 y)	197	45.2/54.8	Serum Plasma Urine Red blood cells	169 552 1,942 137	−80°C/−20°C −20°C −80°C/−20°C −20°C
Hotspot study: Menen	2011	Adolescents (14–15 y)	199	57.3/42.7	Serum Plasma Urine Red blood cells	177 585 2331 195	−80°C/−20°C −20°C −80°C/−20°C
Hotspot study: Ghent canal zone	2013	Adolescents (14–15 y)	200	50.5/49.5	Peripheral blood Peripheral blood cells Serum Plasma Urine EBC^*^	789 249 180 94 564 752	−80°C/−20°C −80°C −80°C −80°C −80°C −80°C

* *EBC, exhaled breath condensate*.

### Flemish Environment and Health Study (FLEHS)

On behalf of the Flemish government, the Center of Expertise on Environment and Health has studied the human exposure and health effects of environmental pollutants by conducting 5 years human biomonitoring campaigns in Flanders (3, 9, 15). The research consortium, a collaborative effort of VITO, the Provincial Institute for Hygiene (PIH) and teams of the five Flemish universities, has applied human biomonitoring to detect the levels and the effects of environmental pollutants in babies, adolescents and adults. Four cycles of human biomonitoring were conducted up to now.

#### FLEHS I

The first Flemish Environment and Health Study (FLEHS I 2002–2006) included participants belonging to three different age groups (newborns and their mothers, 14–15 year old adolescents and 50–65 year old adults) recruited in eight regions in Flanders with different environmental characteristics. In total, about 1,600 participants per age group participated in the study. The exposure and health effects of ‘classic’ historical pollutants [such as heavy metals, dioxin-like compounds, para-dichlorodiphenyldichloro-ethylene (p,p'-DDE), polycyclic aromatic hydrocarbons (PAHs)] were measured. Blood and urine samples were collected. Any leftover specimens after biomarker analyses were stored in a biorepository. Saliva and blood samples were collected in a subpopulation of the longitudinal birth cohort of FLEHS I at the age of 10 years (4).

#### FLEHS II

The FLEHS II (2007–2011) biomonitoring campaign aimed to set reference values for a broad range of environmental pollutants in three age groups of the general population (10). Participants were recruited across Flanders. In addition to the historical pollutants, a large number of “new” pollutants (including phthalates, brominated flame retardants, musk's, new pesticides,…) were investigated in three age groups (1) a newborn cohort (*n* = 250), (2) 14–15 year old adolescents (*n* = 200), and (3) adults between 20 and 40 years of age (*n* = 200). Moreover, adolescents of 14–15 years were recruited in two industrial hotspot areas in Flanders (Genk, Menen) (*n* = 200 in each hotspot) (11). Field work, chemical analyses, database management, statistical analysis, interpretation, and communication was performed according to the same standards as in the reference population. In FLEHS II, cord blood, blood, and urine samples were collected and analyzed for the pre-defined measurements. A specific plan to store biobank samples was put into practice: small additional volumes of samples were collected and stored in the biorepository.

#### FLEHS III

The third human biomonitoring program FLEHS III (2012–2015) continued to build on the broad basis of the first and second cycle. Flemish reference values both for historical and recent pollutants were determined in different age groups: pregnant mothers, adolescents of 14–15 year old, adults (50–65 years). Between 200 and 300 study participants per age group were recruited across Flanders. Specific efforts were made for recruitment of participants from different ethnic origin, low income or low education level. Cord blood, blood, urine, and hair samples were collected. In addition, adolescents were studied in one industrial hotspot area (Ghent canal zone). Similar to FLEHS II, additional samples were taken for long-term storage in the biorepository.

#### FLEHS IV

The fourth campaign FLEHS IV (2016–2020) aimed to recruit 600 adolescents aged 14 and 15 years across Flanders from which 200 participated earlier in the newborn campaign of FLEHS I, 14 years ago. FLEHS IV will examine the exposure to environmental pollutants among young people from the general Flemish population. The study will address present-day topics: environmental exposure and health in Flanders in relation to use of space and eco-behavior related to consumption of locally grown or organic food and housing conditions (use of healthy building materials and energy efficiency). New and emerging chemicals were prioritized based on their relevance for assessing exposures from green/gray/blue/agricultural space and eco-behavior. Samples of whole blood, serum, blood RNA, hair, and urine were stored in the biorepository.

### 3xG Study

The 3xG study is a health monitoring pilot study that has been conducted on behalf of the Belgian Agency for Radioactive Waste and Enriched Fossil Materials (NIRAS) and the local partnerships STORA (Dessel) and MONA (Mol) to survey health in relation to life style and environment of children that are born and grow up in 3 Flemish municipalities (Dessel, Mol, and Retie). The 3xG project is programmed as a long term follow up study for children from before birth until the age of 18 years and was initiated in autumn 2009. The project is carried out by the VITO Health team, Provincial Institute of Hygiene of Antwerp and social scientists of the University of Antwerp and collects biomonitoring data on pesticides, heavy metals, substances in consumer goods and on lifestyle. For this study, urine and blood samples of pregnant women and cord blood samples of their babies were collected, analyzed or stored for later analysis. Monitoring newborns from birth and their long term follow up is used as a sentinel for health of the local population. Early warning and sensitive parameters are collected to reflect potential environmental and life style risk factors and to provide advice for improving health. Special focus is on early warnings for obesity, asthma, allergies, growth, and development and heart and vascular diseases. Presently, 300 mothers and their babies are participating in this study. Cord blood, breast milk, blood, plasma, serum, and urine samples were stored in the biorepository. A follow-up of the children at the age of 7 years is planned in 2019–2020.

## DEMOCOPHES

The objective of the European Seventh Framework Programme COPHES (Consortium to Perform Human biomonitoring on a European Scale) was to develop a harmonized approach to conduct human biomonitoring in Europe. In 17 European countries, the biomonitoring guidelines and protocols developed by COPHES were tested in a biomonitoring pilot study DEMOCOPHES (DEMOnstration of a study to COordinate and Perform Human biomonitoring on a European Scale) (12, 13). Mercury in hair and cotinine, phthalate metabolites and cadmium in urine of 1,844 children (5–11 years of age) and their mothers were measured. The Belgian participant population consisted in 129 children aged 6–11 years and their mothers (≤45 years) living in urban or rural areas of Belgium (14). Samples were collected over a 5 months period in 2011–2012. Leftover samples of urine and hair were stored in the biorepository.

## Conclusion

Biobank@VITO has its limitations and strengths. Biobank@VITO is a population biobank and hosts a heterogeneous collection of samples from the general population spread across Flanders and over time and covers different ages. As no continuous monitoring program exist in Flanders the characteristics of the collections are completely dependent on the research question of the biomonitoring study. Over the years, samples have always been collected and stored according to most appropriate conditions at that time with regard to the selected biomarkers. Due to progressive insight into storage conditions and technological developments, some issues must be taken into account when reusing these samples. The quality of the samples collected a long time ago might not be useful to perform all type of newly developed biomarker analyses because of the way they were collected/stored at that time and the uncertainty of biomarker stability over time. However, for specific purposes, these samples are still very valuable. Further, biobank establishment, biobanking of human samples and biobank management and maintenance is costly and involves a significant workload. These expenses have to be taken into account in project application and implementation and have a considerable impact on the study budget.

However, Biobank@VITO is unique in providing a biological archive of human exposure to environmental chemicals in Flanders. The biobank holds great potential for research on the interaction between health, environment and lifestyle to support policy development in the nexus of environment and health. The biobank is a sustainable platform in which the same human samples can be reanalyzed for new technologically advanced exposure and effect biomarkers. The platform will allow to address specific research questions on population health in relation to environmental factors allowing both prospective and retrospective analysis.

## Data Availability Statement

All datasets generated for this study are included in the article/Supplementary Material.

## Author Contributions

GS, VN, and ED designed and coordinated the Flemish HBM studies FLEHS and 3xG. GS, KV, and ED initiated the biobank initiative at VITO. ED, AC, and RV further elaborated the biobank initiative. RV wrote the paper. All authors evaluated and approved the manuscript.

### Conflict of Interest

The authors declare that the research was conducted in the absence of any commercial or financial relationships that could be construed as a potential conflict of interest.
