# Supplementary material for: Biobank@VITO: Biobanking the General Population in Flanders
Source: Front Med (Lausanne). 2020 Feb 14;7:37. doi: 10.3389/fmed.2020.00037 (PMC7033607; doi:10.3389/fmed.2020.00037)
Supplement: Supplementary file 1 [file Data_Sheet_1.PDF]

INFORMED CONSENT

STUDY name

Title of the study: ….
Commissioner: …….
Researcher (s): ….

Introduction

Dear,

We kindly ask you to participate in a medical research entitled …….. You decide whether you want to participate. Before you make the decision, it is important to know more about the research. Therefore, read this information letter carefully.

This information and consent form describes the purpose, the studies, the benefits, risks and inconveniences associated with the study. The alternatives available to you and the right to leave the study at any time are also described below. No promises can be made nor guarantees can be given regarding the results of the study. You have the right to ask questions at any time about the possible and / or known risks of this study.

Aim and description of the study

Complete…………………………………

Duration of the study

The study runs for …….. months.

Course of the study

We ask that you sign up for a … (*frequency*) … survey if you agree to participate in the study and you meet all the conditions for participating in the study.

We ask you:

-*sample type e.g. blood, urine, …. and volume*

-measurements e.g. length, weight,……..

-…..

The investigation takes place at …….

The total duration of the investigation is approximately ….. hour.

The remains of the samples are stored indefinitely in a biobank and can be used in the future to perform new measurements. It is currently not possible to describe all future measurements. We only carry out measurements that fit in with our environmental and health research. Plans for new research will always be submitted to the ethics committee for approval.

Voluntary participation / Withdrawal of participation from the study

You voluntarily participate in this study and you have the right to refuse to participate.

If you agree to participate, you must keep this information folder and you will be asked to sign the attached consent form.

You have the right to cancel your participation in the study at any time, even after you have signed the consent form. You do not have to give a reason for withdrawing your permission to participate. Please report this to us in writing.You can request that all samples that have been collected are destroyed and no longer form part of this study. Your own results will continue to be part of the study unless you explicitly request them to be removed.The withdrawal of your consent, just like not participating in this study, will not result in any disadvantage or loss of benefits.

Liability and insurance

If you experience damage that is related to this research, the damage will be reimbursed by the principal of this study in accordance with the law on experiments on the human person of 7 May 2004. You do not have to prove an error. The client has taken out civil liability insurance to cover the risks and damage that would result from this study.

Possible risks

There is no risk associated with collecting ……. Taking the blood sample can be unpleasant due to the puncture and in the worst case can cause a bruise.

Costs

Participation in the study is completely free. No research is charged to you.

Protection of privacy

We consider your privacy as very important. We process your data in accordance with the new European privacy legislation. Researchers process the information from the questionnaire and the samples. Your name and address will be replaced by a code. If the results of this study are published in a report or scientific journal, your name and address will not be mentioned in any way. All information is treated confidentially in accordance with the law on privacy.

Ethics Committee

This study was assessed by an independent ethics committee, ……. Ethics Committee, which gave a favorable opinion.

Right to information

You have the right to ask the responsible physician or the principal investigator what information is collected about you in the context of the study and what its purpose is. You also have the right to ask the doctor to get access to your personal data and possibly have improvements made to it. These rights are legally determined by the laws and regulations on the protection of privacy. The researchers will inform you when important changes in the procedures, risks or benefits of this study would occur.

We request that any address changes be reported to the research team.

Information about the state of the research can be found at …….

Within the design of the study, you are provided with the medically interpretable results. If you wish, we will provide you with all your personal results. The group results will also be made available on ….. as well as information about any new analyzes and studies.

Statement by the principal investigator:

The principal investigator is responsible for conducting this research program according to the conditions described in this document.

Signature	*Signature*
**Principle Investigator**	**Responsible doctor**
Name……..	*Name:…….*
Institute:…..	*Institute:….*
Address:…….	*Address:……*
Tel………	*Tel:.....*
e-mail:……..	*e-mail:……*

INFORMED CONSENT

Study: *name…..*

Consent form

PLEASE RETURN THIS DOCUMENT

☐ **I have read the information letter and the consent form.**
I understand the possible risks and benefits of this study.
I volunteer to participate in this study

In this study, samples are stored in the biobank. I give permission to perform additional analyzes on the samples. All measurements that are carried out fit within the "Environment and Health" theme. The researchers request permission from the Ethics Committee for each new measurement.

☐ **Yes, I give permission to perform analyzes on the biobank samples after the measurements have been approved by an Ethics Committee.**

Signature responsible doctor *(name)….*
Date: ………/………/………………

Name participant: …………….. **Signature**

Date ………/………/………………

PLEASE FILL IN THE FOLLOWING DATA:

Do you wish to receive a personal result from the measurements in this study?

(Indicate 1 box)

☐ **Yes, I want to receive personal results at my home address. In case of a change of address I will pass on my new address to the research team**.

☐ **No, I do not want to receive personal results.**

INFORMED CONSENT

Study: *name….*

Consent form

THIS DOCUMENT IS INTENDED FOR YOU

☐ **I have read the information letter and the consent form.**
I understand the possible risks and benefits of this study.
I volunteer to participate in this study.

In this study, samples are stored in the biobank. I give permission to perform additional analyzes on the samples. All measurements that are carried out fit within the "Environment and Health" theme. The researchers request permission from the Ethics Committee for each new measurement.

☐ **Yes, I give permission to perform analyzes on the biobank samples after the measurements have been approved by an Ethics Committee.**

Signature responsible doctor (name)….
Date: ………/………/………………

Name participant: …………….. **Signature**

Date ………/………/………………

PLEASE FILL IN THE FOLLOWING DATA:
Do you wish to receive a personal result from the measurements in this study?
(Indicate 1 box**)**

☐ **Yes, I want to receive personal results at my home address. In case of a change of address I will pass on my new address to the research team.**

☐ **No, I do not want to receive personal results.**
